# Association between Eating Away from Home and Hyperuricemia: A Population-Based Nationwide Cross-Sectional Study in China

**DOI:** 10.1155/2019/2792681

**Published:** 2019-10-03

**Authors:** Zifeng Liu, Xiaoting Su, Mianli Xiao, Peien Zhou, Jianwei Guo, Yixiang Huang, Yiqiang Zhan

**Affiliations:** ^1^Department of Clinical Data Center, The 3rd Affiliated Hospital, Sun Yat-Sen University, Guangzhou 510080, China; ^2^Department of Health Policy and Management, School of Public Health, Sun Yat-Sen University, Guangzhou 510080, China; ^3^Department of Medical Epidemiology and Biostatistics, Karolinska Institutet, Stockholm 17177, Sweden

## Abstract

Hyperuricemia (HU) is a risk factor for different kinds of chronic noncommunicable diseases, and eating away from home (EAFH) may play an important role in their development, which has been ignored greatly so far. This study aimed to investigate the association between EAFH and HU in different models. A cross-sectional study involving 8,322 participants of the China Health and Nutrition Survey (CHNS) was conducted. Logistic regression models were used to analyze the data. We found that participants who consumed more away-from-home food had a higher risk for HU, and the adjusted odds ratio (aOR) and 95% confidence interval (CI) (for each increment in grades of EAFH) were 1.11 (1.02, 1.20) in a multiadjusted model (adjusted for age, gender, province, net individual income, body mass index, smoking, leisure-time physical activities, energy intake, and sleep duration). As for stratified analyses, the aOR (95% CI) of EAFH was 1.12 (1.01, 1.24) for men and 1.06 (0.92, 1.21) for women. Similar results can be found in the middle-aged and obese population, with aOR (95% CI) of EAFH as 1.17 (1.05, 1.30) and 1.15 (1.03, 1.29), respectively. In conclusion, EAFH is positively associated with the prevalence of HU.

## 1. Introduction

The accumulated research studies have demonstrated that hyperuricemia (HU) plays an important role in the development of many chronic noncommunicable diseases, such as metabolic syndrome, chronic kidney disease, gout, and cancer [1–5]. The reported prevalence of HU varies from 2.6% to 36.0% in different countries, which has rapidly increased worldwide in the past few decades [6, 7]. A number of epidemiological studies on HU have illustrated that food ingredients are significantly associated with HU after adjusting for other risk factors, including age, gender, physical activities (PA), and body mass index (BMI) [8–11].

In our modern time-pressed society, convenience becomes a way of life for a lot of individuals. Eating away from home (EAFH) is an increasingly important part of the people's diet. However, evidences showing that EAFH will have an inappropriate portion of nutrient intake and poor diet quality have been increasing [12–14]. Previous studies showed that EAFH, which led to high intake of calories, saturated fat, cholesterol, and minerals like sodium and calcium, as well as the lack of fruit and vegetable consumption, might be a risk factor for different kinds of chronic diseases, such as heart diseases, obesity, diabetes, and hypertension [15, 16]. However, whether EAFH is a risk factor for HU after adjusting for other confounders remains unclear to date, and the impact of EAFH on HU has not been fully explored.

In recent years, with the development of social economy, EAFH, an increasingly integral practice in Chinese diet, has become one of the most common ways of food consumption in China. A synchronous ascending trend between the prevalence of EAFH and HU was observed in recent years [17–19]. The rate of EAFH consumption increased from 16.0% to 18.3% in urban areas and from 6.1% to 11.1% in rural China from 2004 to 2011 [17]. As for the prevalence of HU, the adjusted prevalence of hyperuricemia among Chinese adults in 2009–2010 was 8.4% [18]. The pooled prevalence of hyperuricemia was 13.3%, which greatly varied and appeared to be increasing [19]. Furthermore, a relatively larger burden of hyperuricemia is expected in China as compared with other developing countries for a series of social trends [20]. Yet, regarding away-from-home foods, the public focuses more on the prevention of contaminants and food-borne diseases instead of their contribution to HU [21]. To date, no nationwide population-based studies have been conducted in China to explore the relationship between EAFH and the risk of HU. Therefore, we conducted this study.

This study aimed to explore the association between EAFH and HU based on the following hypothesis: EAFH is associated with the risk of HU in Chinese adult population. Furthermore, some specific suggestions were also provided for the public to prevent HU, such as diet adjustment, i.e., increasing vegetable intake and reducing fat consumption.

## 2. Materials and Methods

### 2.1. Study Population

**Figure 1:**
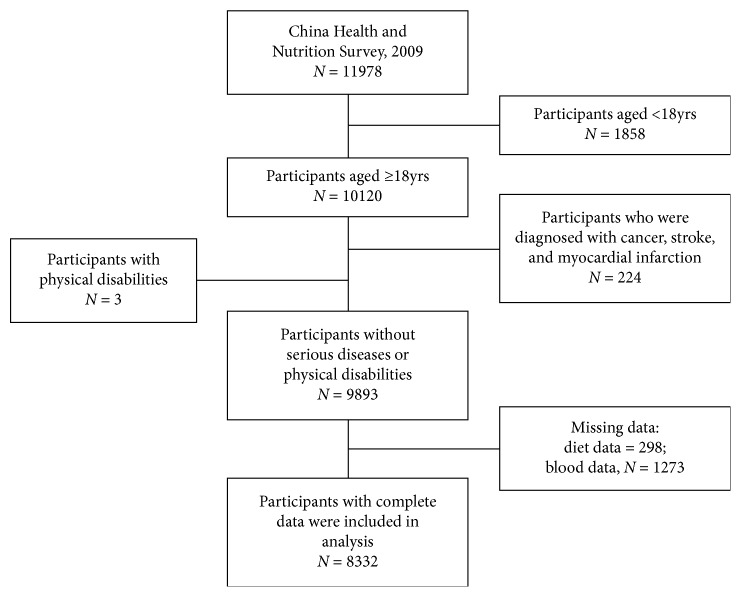
Flowchart of study.

We used data from the China Health and Nutrition Survey (CHNS), a population-based observational cohort study in 9 different provinces (Heilongjiang, Liaoning, Shandong, Henan, Jiangsu, Hubei, Guizhou, Hunan, and Guangxi) over China. Details of study design of the CHNS have been described in previous research [22]. As shown in Figure 1, data were collected from the 2009 wave of CHNS, in which 11,978 participants participated in the survey. During this survey, fasting blood samples were collected, and detection was conducted. Adult population aged ≥18 years were included in our analyses (*n* = 10,120). A total of 224 participants with serious diseases (i.e., cancer, stoke, and myocardial infarction), 3 with body disability that affects going out, 1,273 with no blood sample collection or assessment on serum uric acid (SUA), and 298 without diet information were excluded. Finally, 8,322 participants (3,878 men and 4,444 women) were included in analyses.

### 2.2. Laboratory Examinations

Blood samples (12 mL) were collected through venipuncture in the morning after an overnight fasting for at least 12 h. Laboratory methods used to analyze biomarkers are described in a previous study [23]. SUA concentrations were detected by an enzymatic colorimetric method using a Hitachi 7600 automated analyzer (Hitachi Inc., Tokyo, Japan) and with determiner regents (Randox Laboratories Ltd., Crumlin, UK) [23].

### 2.3. Diet Data

As for the diet data, researchers used a combination of 3 successive 24 h dietary recalls on the individual level alone with food inventory at the family level during a 3-day period to gather dietary information, randomly starting any day of the week [23]. For the 3-day 24 h recalls, all types and amounts of foods, dining types, and places of food preparation of every food item were recorded by trained interviewers. Additional information about the dietary data collection has been described in previous studies in detail [24, 25]. A Chinese food composition table (2004) was used to evaluate nutrient intakes and then to calculate the average total energy and fat intakes of every participant for 3 days [26]. In addition, the average fruit and vegetable intakes were also calculated based on a food grouping system developed for the CHNS, including 162 fruit and 256 vegetable items [27].

### 2.4. Exposure and Outcome

In our study, we focused on EAFH as the exposure. EAFH is defined as the consumption of foods that were not cooked at home (whether consumed at or away from home) during the survey period and then categorized into nonconsumers, occasional consumers (>0 and < 1 meal/day), and frequent consumers (≥1 meal/day). The prevalence of HU served as the outcome of this study. According to previous HU guidelines, SUA concentrations of ≥7 mg/dL for men and ≥6 mg/dL for women are defined as HU [1].

### 2.5. Covariates

We chose the covariates listed below based on previous epidemiologic analyses [28, 29]. At each wave of survey, participants' age, gender, province (including Guangxi, Guizhou, Henan, Heilongjiang, Hubei, Hunan, Jiangsu, Liaoning, and Shandong; province was coded with dummy variables), and PA data were collected from a self-reported 7-day PA recalls. PA was calculated using hours spent in each activity multiplied by metabolic equivalents (MET) for that activity and defined leisure-time PA as the sum of MET *∗* h for all leisure-time sports including dancing, gymnastics, track-and-field sports, ball sports, swimming, and other sports. Sleep duration was evaluated using self-reported questionnaires, and responses on sleep duration ranged from 1 to 18 h. Age was categorized into “young (18–40 years),” “middle aged (41–65 years),” and “old age (66 years, +∞).” Gender was categorized into “female” and “male.” Smoking was categorized into “nonsmoking” and “smoking.” Alcohol drinking was categorized into “yes” and “no.” Weight and height of participants were measured with light clothing and without shoes and then calculated into BMI as weight (kg)/height (m^2^).

### 2.6. Statistical Analysis

In descriptive analyses, characteristics of included participants were examined and categorized according to the presence of HU and non-HU, age, gender, province, net individual income, smoking, alcohol drinking, away-from-home eating categories, BMI, leisure-time PA, and sleep duration (Table 1). We conducted logistic regression models to examine the associations of EAFH with the presence of HU in 2009. In this analysis, EAFH was enrolled into logistic regression as an ordered multicategorical variable (0 = nonconsumers, 1 = occasional consumers (>0 and < 1 meal/day), and 2 = frequent consumers (≥1 meal/day)) and then the OR and 95% CI for the increment of EAFH (0–1 or 1–2) were calculated. Moreover, four models were fit in the analysis: model 1 (no adjustment), model 2 (adjusted for age and gender), model 3 (adjusted for variables in model 2 and individual income, BMI, smoking, leisure-time physical activities, provinces, and sleep duration), and model 4 (adjusted for variables in model 3 and energy, drinking, and vegetable and fruit intakes). Stratified analyses by gender (female/male), age categories (youth/middle aged/old aged), and BMI (<24/≥24) were conducted by including interaction terms to examine whether the association between EAFH and HU was different between these factors. We also examined the difference on diet (including intake of total energy, fat, vegetables and fruits and frequency of tea and alcohol drinking) between EAFH categories using the Kruskal–Wallis test because food intakes were not normally distributed.

### 2.7. Sensitivity Analysis

In our study, a total of 1,571 individuals without accurate data were excluded, and estimates obtained from this “complete-case” (CC) analysis may lead to selection bias if the excluded ones are systematically different from those included. Inverse probability weighting (IPW) is a common method to reduce this bias. We code the censor data with dummy variables and provided each uncensored data a weight to correct possible selection biases. Details are described in the relevant study [30]. Therefore, we applied IP estimation in model 3 and then the OR and 95% CI were estimated using this model. At the same time, other sensitivity analysis was performed by recoding occasional consumers and frequent consumers into one group and nonconsumers as the control group to verify the consistency of results. These analyses were conducted using R (version 3.5.1); *p* values < 0.05 were used to indicate significance.

## 3. Results

### 3.1. Characteristics of the Study Population

Characteristics of participants (total, HU, and non-HU population) in this study are presented in Table 1. The prevalence of HU is 15.4% in the total population (11.0% for female and 20.4% for male). Compared with participants without HU, those with HU were more likely to be male (*P* < 0.01), are older (*P* < 0.01), have higher BMI (*P* < 0.05), have higher individual income (*P* < 0.05), tended to smoke (*P* < 0.01), consumed alcohol (*P* < 0.01), have higher EAFH frequency (*P* < 0.01), and more leisure-time physical activities (*P* < 0.05) but showed no difference in sleeping time. The prevalence of HU also varied in provinces (*P* < 0.01).

### 3.2. Association between EAFH and HU in Different Models

**Figure 2:**
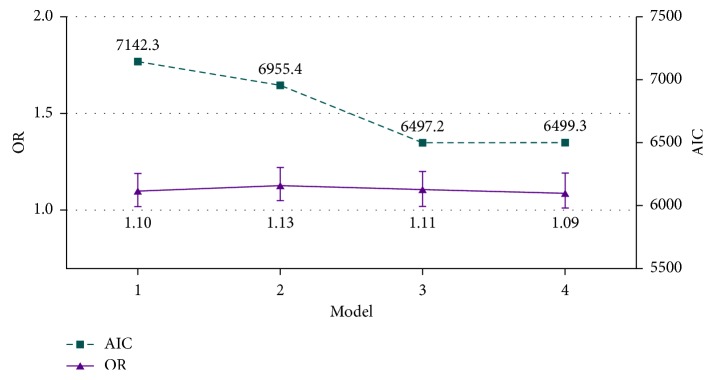
Trend of OR and AIC according to the variation of model complexity.

Logistic regression results in different models are shown in Table 2. The OR and 95% CI (for each increment in grades of EAFH) were 1.10 (1.02, 1.19), 1.13 (1.05, 1.22), 1.11 (1.02, 1.20), and 1.09 (1.01, 1.19) in models 1 to 4, respectively. Compared with model 3, many other covariates were adjusted in model 4 (covariates in model 3 and add energy, vegetable, and fruit intake and drinking), and the OR was 1.09. OR and AIC trends (index of goodness fit of model) according to the variation of model complexity are shown in Figure 2. The AIC of model quickly decreased in models 1 to 3 but increased in model 4, which means the fitting of model has become stable at model 3.

As for stratified analyses, the OR and 95% CI of EAFH were 1.06 (0.92, 1.21) among women and 1.12 (1.01, 1.24) among men in model 3 (multiadjusted model). When stratified by age stages (young, middle, and old aged) and BMI (<24 and ≥ 24), people who consumed away-from-home foods were found to have a higher prevalence of HU, which could only be found in the middle-aged population (1.17 95% CI: 1.05, 1.30) and population with BMI of ≥24 (1.15 95% CI: 1.03, 1.29) in model 3.

### 3.3. Difference in Nutritional Ingredients between Nonconsumers, Occasional Consumers, and Frequent Consumers

The comparison of nutritional ingredients between nonconsumers, occasional consumers, and frequent consumers is shown in Supplemental [Supplementary-material supplementary-material-1]. We found that compared with nonconsumers, participants who consumed away-from-home food consumed more energy, fat, protein, and alcohol and less vegetables and tea (all *p* value <0.05). The results of diet comparison stratified by gender, age, and BMI are shown in Supplemental Tables [Supplementary-material supplementary-material-1]–[Supplementary-material supplementary-material-1], and the difference and similar tendency on food intakes could still be found after stratification.

### 3.4. Sensitivity Analysis

The aOR and 95% CI estimated by IPW (subjects with missing data enrolled) were 1.02 (1.01, 1.04) for model 2, 1.01 (1.002, 1.023) for model 3, and 1.01 (1.002, 1.023) for model 4. All *p* values were <0.05. Moreover, by coding occasional consumers and frequent consumers into one group and nonconsumers as the control group, we found that the aOR and 95% CI (1.16 (1.02, 1.32)) remained statistically significant. These results suggested the robustness of the model.

## 4. Discussion and Conclusion

HU is a metabolic disorder that seriously endangers health. In this study, the prevalence of HU in the Chinese population is at a high level (15.4%), which is higher than the one in previous nationwide meta-analysis (13.3%). Our results show a lower prevalence of HU than developed countries such as the United State (21.4%) and Japan (25.8%) but higher than other developing countries such as Thailand (10.6%) and Turkey (12.1%) [19]. These results show that HU has become a major disease endangering the health of Chinese people. In this study, we found that EAFH is associated with HU in China. After adjusting confounding factors and sensitivity analysis, the correlation still exists. Stratified by age, gender, and BMI, we further found one more important result: obesity (BMI ≥ 24), male, and middle-aged people who eat out are at higher risks of HU. To our knowledge, this study is the first to describe the relationship between EAFH and HU in China, which may build a scientific base to promote changes of people's lifestyle and behavior.

With the development of the Chinese economy and the faster pace of life, health risks related to eating become an important public health problem. This study found that the rate of eating out in China is as high as 37.4%, which is similar to that in other studies [26, 27]. Previous studies have shown that eating out can increase the risk of many diseases [19, 31]. However, research on the relationship between EAFH and HU remains to be determined. This study fills this gap. Our results show that eating out may increase the risk of HU (OR = 1.11, in model 3) for the total population. Among the 41–65-year-olds, male, and obese people, the risk increased by 17.0%, 12.0%, and 15.0%, respectively. The increase in range of risk was higher than that of the general population, showing that EAFH requires special attention for these people.

We further explored the nutritional mechanism of the fact that EAFH is associated with HU. There was a significant difference in the dietary composition between people who ate out and at home. Our results show that EAFH is characterized by high fat, high protein, low carbohydrate, less vegetable intake and more alcohol and tea intake. These results are consistent with several studies [8–11, 32, 33]. Among the middle-aged, male, and obesity subgroups, the difference of food ingredients in eating out has the same abovementioned characteristics. Our findings are consistent with that of other researches, which also show that high intakes of purine, protein, sugar, fat, alcohol, and sugary drinks are risk factors for HU [12, 15, 16]. Our results showed that, after adjusting possible confounding factors, the association between EAFH and HU remained significant among male people, but the similar result could not be seen in the female subgroup. We think that the reason can be associated with the genetic and hormonal difference between genders. Kolz et al. found that, among women, the minor allele for rs734553 in SLC2A9 contributed more in lowering the uric acid levels, and the minor allele of rs2231142 in ABCG2 had a stronger power in elevating uric acid levels in men compared to women [34]. As for hormones, Hak et al. illustrated that female hormones like estrogens and progesterone had impacts on the kidney and helped clean urate more effectively [35]. These findings provide explanation on the biological mechanism of the gender difference in the risk of HU, but the real reason needed further exploration. Considering that dietary factors mentioned above may be major confounders of the study, we used models to adjust their impacts on the result. After adjusting these food factors, the relationship between EAFH and HU remains significant, which suggested that EAFH is associated with HU.

Previous studies have elaborated on the impact of specific foods on the risk of HU [12–14, 32]; however, little attention has been paid to an individual's eating habit, and the guiding significance is limited. This study focuses on the impact of EAFH on HU, which is of great significance to guide behavioral changes. Our findings propose dietary warnings for high-risk groups, and we advise them to pay more attention to their food matching, such as increasing vegetable intake and reducing fat consumption. The findings can provide references for government's health promotion policies and promote people to pay attention to their health, optimize the dietary structure, and enhance the ability to control their own health. The results of this study revealed that the status of eating out is similar to that in many developing countries; therefore, the health promotion significance might be further generalized and promoted.

The advantage of this study is that it is the pioneering study to qualify and quantify the relationship between EAFH and the risk of HU. It also elucidates three specific high-risk subpopulations in China, i.e., middle-aged, male, and obese people. In addition, this study benefits from a large sample size of a population-based national study with good representativeness.

There are also some limitations in this study. Firstly, although the possibility of inversion of causality in this study is low, its proof power remains inferior to that of a cohort study. Secondly, there may be confounding factors that we were not able to identify. Moreover, as an important factor associated with HU, unhealthy habits in home prepared food were not included in our models, which might lead to inevitable confounding in our study. Therefore, the findings should be confirmed by conducting prospective cohort studies, especially on whether the frequency of dining out of young people is a risk factor of HU in their old age, which we find to be another valuable study to be carried out in the future.

In conclusion, EAFH was positively associated with HU, which may result from inappropriate proportion of nutrient intake. People who eat away from home have higher risk of HU, especially the middle-aged, male, and obese ones. These findings can provide references for government's health promotion policies and enhance individuals' ability to control their own health.

## Figures and Tables

**Table 1:** Characters of total population and hyperuricemia population and nonhyperuricemia population.

Characters	Total	Hyperuricemia	Nonhyperuricemia	*p* value
	8322	1280 (15.4)	7042 (84.6)	
Age, *N* (%)				<0.01
Youth	2298 (27.6)	286 (22.3)	2012 (28.6)	
Middle age	4682 (56.3)	719 (56.2)	3963 (56.3)	
Old age	1342 (16.1)	275 (21.5)	1067 (15.2)	
Gender, *N* (%)				<0.01
Female	4444 (53.4)	489 (38.2)	3955 (56.2)	
Male	3878 (46.6)	791 (61.8)	3087 (43.8)	
Province, *N* (%)				<0.01
Guangxi	1023 (12.3)	221 (17.3)	802 (11.4)	
Guizhou	757 (9.1)	161 (12.6)	596 (8.5)	
Henan	943 (11.3)	78 (6.1)	865 (12.3)	
Heilongjiang	859 (10.3)	141 (11.0)	718 (10.2)	
Hubei	897 (10.8)	135 (10.5)	762 (10.8)	
Hunan	1054 (12.7)	153 (11.9)	901 (12.8)	
Jiangsu	1074 (12.9)	177 (13.8)	897 (12.7)	
Liaoning	785 (9.4)	135 (10.5)	650 (9.2)	
Shandong	931 (11.2)	80 (6.2)	851 (12.1)	
Net individual income, yuan/year				<0.05
<8000	2190 (26.3)	262 (20.5)	1928 (27.4)	
8000–15000	1797 (21.6)	287 (22.4)	1510 (21.4)	
15000–20000	2871 (34.5)	447 (34.9)	2424 (34.4)	
≥20000	1464 (17.6)	284 (22.2)	1180 (16.8)	
Smoking, *N* (%)				<0.01
Nonsmoking	5761 (69.2)	786 (61.4)	4975 (70.6)	
Smoking	2561 (30.8)	494 (38.6)	2067 (29.4)	
Alcohol drinking, *N* (%)				<0.01
Yes	2701 (32.5)	560 (43.8)	2141 (30.4)	
No	5621 (67.5)	720 (56.3)	4901 (69.6)	
Away-from-home eating, *N* (%)				<0.01
Nonconsumers	5209 (62.6)	757 (59.1)	4452 (63.2)	
Occasional consumers (>0 and <1 meal/d)	1622 (19.5)	273 (21.3)	1349 (19.2)	
Frequent consumers (≥1 meal/d)	1491 (17.9)	250 (19.5)	1241 (17.6)	
Body mass index (BMI), kg/m^2^ (IQR)	23.2 (21.0–25.5)	24.6 (22.3–27.1)	22.9 (20.8–25.1)	<0.05
Leisure-time physical activities, MET-h/day (IQR)	8.92 (4.96–14.12)	8.32 (4.60–13.41)	9.03 (5.03–14.19)	<0.05
Sleeping time, h/d (IQR)	8.0 (7.00–8.00)	8.0 (7.00–8.00)	8.0 (7.00–8.00)	0.17

MET: metabolic equivalent; IQR: interquartile range.

**Table 2:** Association between EAFH and HU in China, OR (95% CI).

Population	Model 1^a^	Model 2^b^	Model 3^c^	Model 4^d^
Total population	1.10 (1.02, 1.19)^#^	1.13 (1.05, 1.22)^#^	1.11 (1.02, 1.20)^#^	1.09 (1.01, 1.19)^#^
Gender				
Male	1.17 (1.06, 1.28)^#^	1.16 (1.05, 1.28)^#^	1.12 (1.01, 1.24)^#^	1.11 (1.00^*∗*^, 1.24)^#^
Female	0.94 (0.83, 1.07)	1.06 (0.93, 1.21)	1.06 (0.92, 1.21)	1.03 (0.90, 1.19)
Age				
Youth	1.00 (0.87, 1.16)	0.96 (0.82, 1.12)	0.96 (0.80, 1.14)	0.94 (0.78, 1.11)
Middle age	1.22 (1.10, 1.34)^#^	1.19 (1.08, 1.31)^#^	1.17 (1.05, 1.30)^#^	1.15 (1.03, 1.29)^#^
Old age	1.14 (0.93, 1.38)	1.14 (0.93, 1.39)	1.10 (0.89, 1.35)	1.09 (0.88, 1.35)
BMI				
BMI < 24 kg/m^2^	0.99 (0.89, 1.11)	1.07 (0.95, 1.20)	1.05 (0.93, 1.18)	1.04 (0.92, 1.17)
BMI ≥ 24 kg/m^2^	1.20 (1.08, 1.33)^#^	1.16 (1.04, 1.29)^#^	1.15 (1.03, 1.29)^#^	1.14 (1.01, 1.27)^#^

Model 1^a^: without adjusting for any covariate, unadjusted OR; model 2^b^: model adjusted for age and gender, adjusted OR; model 3^c^: model adjusted for age, gender, province, net individual income, BMI, smoking, and leisure-time physical activities, and sleep duration, adjusted OR; model 4^d^: model adjusted for covariates in model 3 and energy, vegetable, fruit intakes and alcohol drinking, adjusted OR; ^*∗*^CL 1.002 gives 1.00 when rounded off to 2 decimal places; ^#^p for trend, *p* < 0.05.

## Data Availability

Data of CHNS can be viewed and obtained from the following website: https://www.cpc.unc.edu/projects/china.

## Supplementary Materials

Supplementary Materials Refer Table S1 in the Supplementary Material for the comparison of nutritional ingredients among the three groups. The stratified comparison of nutritional ingredients among the three groups is shown in Tables S2–S4 in the Supplementary Material, respectively.
