# Supplementary material for: Association between Eating Away from Home and Hyperuricemia: A Population-Based Nationwide Cross-Sectional Study in China
Source: Biomed Res Int. 2019 Oct 3;2019:2792681. doi: 10.1155/2019/2792681 (PMC6794973; doi:10.1155/2019/2792681)
Supplement: Supplementary Materials — Refer Table S1 in the Supplementary Material for the comparison of nutritional ingredients among the three groups. The stratified comparison of nutritional ingredients among the three groups is shown in Tables S2–S4 in the Supplementary Material, respectively. [file 2792681.f1.pdf]

Supplemental Table 1. Comparison of diet between three groups of away-from-home eating

Diet, median (IQR)	Nonconsumer	Occasional consumer	Frequent consumer	*P-value*
N	5209 (62.6)	1622 (19.5)	1491 (17.9)	
Energy intakes,kcal/d	2062 (1667-2509)	2091 (1716-2510)	2120 (1712-2546)	**0.011**
Fat intakes, g/d	64.7 (46.8-89.1)	76.2 (55.0-99.8)	77.6 (55.6-105.3)	**<0.001**
Protein intakes, g/d	180.6 (144.4-226.7)	197.6 (159.2-247.0)	204.5 (161.0-252.9)	**<0.001**
Carbohydrate intakes, g/d	874.1 (692.7-1102.2)	803.7 (651.0-1010.7)	816.9 (638.3-994.0)	**<0.001**
Vegetables intakes, g/d	316.7 (220.0-433.3)	296.7 (210.0-383.3)	286.7 (183.3-400.0)	**<0.001**
Tea drinking, N (%)				**<0.001**
Non-drinking	3518 (67.5)	968 (59.7)	913 (61.2)	
Few times per month	135 (2.6)	49 (3.0)	43 (2.9)	
Few times per week	354 (6.8)	143 (8.8)	121 (8.1)	
Almost every day	1202 (23.1)	462 (28.5)	414 (27.8)	
Alcohol drinking, N (%)				**<0.001**
Non-drinking	3688 (70.8)	1030 (63.5)	903 (60.6)	
No more than once a month	178 (3.4)	91 (5.6)	92 (6.2)	
Once or twice a month	334 (6.4)	130 (8.0)	132 (8.9)	
Once or twice a week	354 (6.8)	134 (8.2)	137 (9.2)	
3-4 times a week	190 (3.7)	69 (4.3)	78 (5.2)	
Almost every day	465 (8.9)	168 (10.4)	149 (10.0)	

IQR: interquartile range;

Supplemental Table 2. Comparison of diet between three groups of away-from-home eating stratified by gender

Diet, median (IQR)	Nonconsumer	Occasional consumer	Frequent consumer	*P-value*
**Male (3878)**				
N	2352 (60.6)	743 (19.2)	783 (20.2)	
Energy intakes,kcal/d	2250 (1852-2704)	2337 (1893-2729)	2334 (1932-2764)	**0.019**
Fat intakes, g/d	69.6 (50.7-95.4)	83.0 (60.4-107.4)	82.0 (59.9-113.4)	**<0.001**
Protein intakes, g/d	197.6 (158.8-245.1)	217.6 (174.6-271.3)	224.6 (178.0-274.8)	**<0.001**
Carbohydrate intakes, g/d	967.7 (756.2-1206.9)	899.1 (713.1-1113.2)	897.5 (716.0-1072.4)	**<0.001**
Vegetables intakes, g/d	336.2 (244.0-448.6)	309.5 (231.3-409.8)	298.5 (208.1-401.3)	**<0.001**
Tea drinking, N (%)				**<0.001**
Non-drinking	1347 (57.3)	366 (49.3)	385 (49.2)	
Few times per month	75 (3.2)	27 (3.6)	27 (3.4)	
Few times per week	197 (8.4)	79 (10.6)	76 (9.7)	
Almost every day	733 (31.2)	271 (36.5)	295 (37.7)	
Alcohol drinking, N (%)				**<0.001**
Non-drinking	1020 (43.4)	262 (35.3)	276 (35.2)	
No more than once a month	127 (5.4)	53 (7.1)	56 (7.2)	
Once or twice a month	291 (12.4)	99 (13.3)	111 (14.2)	
Once or twice a week	308 (13.1)	119 (16.0)	126 (16.1)	
3-4 times a week	174 (7.4)	59 (7.9)	74 (9.5)	
Almost every day	432 (18.4)	151 (20.3)	140 (17.9)	
**Female (4444)**				
N	2857 (64.3)	879 (19.8)	708 (15.9)	
Energy intakes,kcal/d	1903 (1556-2300)	1943 (1586-2260)	1920 (1547-2257)	0.058
Fat intakes, g/d	168.7 (135.3-211.6)	182.2 (146.6-228.3)	183.45 (146.5-225.1)	**<0.001**
Protein intakes, g/d	806.7 (648.5-1009.8)	740.6 (612.7-907.1)	721.01 (578.0-887.2)	**<0.001**
Carbohydrate intakes, g/d	268.9 (216.2-336.6)	246.9 (204.2-302.4)	240.3 (192.7-295.7)	**<0.001**
Vegetables intakes, g/d	306.8 (220.6-412.9)	279.7 (207.7-361.7)	276.0 (192.0-371.1)	**<0.001**
Tea drinking, N (%)				**<0.001**
Non-drinking	2171 (76.0)	602 (68.5)	528 (74.6)	
Few times per month	60 (2.1)	22 (2.5)	16 (2.3)	
Few times per week	157 (5.5)	64 (7.3)	45 (6.4)	
Almost every day	469 (16.4)	191 (21.7)	119 (16.8)	
Alcohol drinking, N (%)				**<0.001**
Non-drinking	2668 (93.4)	768 (87.4)	627 (88.6)	
No more than once a month	51 (1.8)	38 (4.3)	36 (5.1)	
Once or twice a month	43 (1.5)	31 (3.5)	21 (3.0)	
Once or twice a week	46 (1.6)	15 (1.7)	11 (1.6)	
3-4 times a week	16 (0.6)	10 (1.1)	4 (0.6)	
Almost every day	33 (1.2)	17 (1.9)	9 (1.3)	

IQR: interquartile range;

Supplemental Table 3. Comparison of diet between three groups of away-from-home eating stratified by age

Diet, median (IQR)	Nonconsumer	Occasional consumer	Frequent consumer	*P-value*
**Youth (18-40y), N=2298**				
N	1262 (54.9)	479 (20.8)	557 (24.2)	
Energy intakes,kcal/d	2112 (1725-2534)	2154 (1786-2603)	2096 (1721-2530)	0.171
Fat intakes, g/d	64.3 (47.0-87.0)	73.8 (55.8-98.7)	76.1 (53.3-98.4)	**<0.001**
Protein intakes, g/d	188.3 (150.7-228.2)	211.1 (170.7-257.9)	210.7 (166.0-258.4)	**<0.001**
Carbohydrate intakes, g/d	926.1 (739.7-1148.6)	850.2 (690.1-1048.7)	832.7 (656.0-1007.5)	**<0.001**
Vegetables intakes, g/d	310.6 (224.0-421.1)	291.4 (212.5-377.0)	279.5 (192.6-375.8)	**<0.001**
Tea drinking, N (%)				**<0.001**
Non-drinking	925 (73.3)	313 (65.3)	379 (68.0)	
Few times per month	41 (3.2)	17 (3.5)	21 (3.8)	
Few times per week	93 (7.4)	56 (11.7)	44 (7.9)	
Almost every day	203 (16.1)	93 (19.4)	113 (20.3)	
Alcohol drinking, N (%)				**<0.001**
Non-drinking	883 (70.0)	306 (63.9)	328 (58.9)	
No more than once a month	72 (5.7)	35 (7.3)	52 (9.3)	
Once or twice a month	110 (8.7)	47 (9.8)	62 (11.1)	
Once or twice a week	93 (7.4)	49 (10.2)	60 (10.8)	
3-4 times a week	50 (4.0)	20 (4.2)	31 (5.6)	
Almost every day	54 (4.3)	22 (4.6)	24 (4.3)	
**Middle age (41-65y), N=4682**				
N	2965 (63.3)	910 (19.4)	807 (17.2)	
Energy intakes,kcal/d	2121 (1715-2576)	2105 (1756-2486)	2174 (1766-2608)	0.104
Fat intakes, g/d	67.1 (48.6-91.4)	78.6 (56.7-100.7)	80.6 (58.3-111.5)	**<0.001**
Protein intakes, g/d	185.3 (149.9-232.3)	197.5 (160.2-245.3)	206.0 (163.1-253.7)	**<0.001**
Carbohydrate intakes, g/d	905.8 (715.9-1125.2)	802.1 (659.5-1003.7)	829.7 (647.6-1000.2)	**<0.001**
Vegetables intakes, g/d	324.9 (235.6-439.0)	297.8 (218.6-393.9)	295.2 (210.9-402.6)	**<0.001**
Tea drinking, N (%)				**<0.001**
Non-drinking	1971 (66.5)	523 (57.5)	455 (56.4)	
Few times per month	71 (2.4)	29 (3.2)	20 (2.5)	
Few times per week	212 (7.2)	73 (8.0)	71 (8.8)	
Almost every day	711 (24.0)	285 (31.3)	261 (32.3)	
Alcohol drinking, N (%)				**<0.001**
Non-drinking	2045 (69.0)	559 (61.4)	468 (58.0)	
No more than once a month	90 (3.0)	51 (5.6)	36 (4.5)	
Once or twice a month	192 (6.5)	72 (7.9)	63 (7.8)	
Once or twice a week	219 (7.4)	75 (8.2)	74 (9.2)	
3-4 times a week	123 (4.1)	43 (4.7)	46 (5.7)	
Almost every day	296 (10.0)	110 (12.1)	120 (14.9)	
**Old age (≥66y), N=1342**				
N	982 (73.2)	233 (17.4)	127 (9.5)	
Energy intakes,kcal/d	1806 (1480-2221)	1837 (1424-2328)	1847 (1563-2192)	0.758
Fat intakes, g/d	58.8 (40.3-84.4)	71.4 (47.7-97.0)	68.0 (44.9-92.5)	**<0.001**
Protein intakes, g/d	159.0 (127.1-199.4)	170.8 (132.3-213.2)	171.5 (144.8-212.0)	**<0.001**
Carbohydrate intakes, g/d	745.9 (592.2-942.7)	704.6 (541.6-884.1)	677.5 (566.1-843.4)	**<0.001**
Vegetables intakes, g/d	313.4 (230.5-421.1)	289.0 (203.2-368.2)	259.3 (192.7-359.8)	**<0.001**
Tea drinking, N (%)				**<0.001**
Non-drinking	622 (63.3)	132 (56.7)	79 (62.2)	
Few times per month	23 (2.3)	3 (1.3)	2 (1.6)	
Few times per week	49 (5.0)	14 (6.0)	6 (4.7)	
Almost every day	288 (29.3)	84 (36.1)	40 (31.5)	
Alcohol drinking, N (%)				**<0.001**
Non-drinking	760 (77.4)	165 (70.8)	107 (84.3)	
No more than once a month	16 (1.6)	5 (2.1)	4 (3.1)	
Once or twice a month	32 (3.3)	11 (4.7)	7 (5.5)	
Once or twice a week	42 (4.3)	10 (4.3)	3 (2.4)	
3-4 times a week	17 (1.7)	6 (2.6)	1 (0.8)	
Almost every day	115 (11.7)	36 (15.5)	5 (3.9)	

IQR: interquartile range;

Supplemental Table 4. Comparison of diet between three groups of away-from-home eating stratified by

Diet, median (IQR)	Nonconsumer	Occasional consumer	Frequent consumer	*P-value*
**BMI < 24, N=5060**				
N	3205 (63.3)	950 (18.8)	905 (17.9)	
Energy intakes,kcal/d	2057 (1660-2514)	2057 (1674-2503)	2090 (1681 -2489)	0.812
Fat intakes, g/d	63.9 (46.6-88.8)	73.9 (53.4-97.6)	76.6 (54.8-102.8)	**<0.001**
Protein intakes, g/d	178.5 (142.6-223.3)	194.1 (155.4-241.4)	200.5 (157.6-249.7)	**<0.001**
Carbohydrate intakes, g/d	877.5 (693.7-1112.2)	794.6 (642.6-1010.4)	798.4 (632.1-982.5)	**<0.001**
Vegetables intakes, g/d	318.3 (232.7-432.2)	297.3 (216.7-388.7)	285.5 (200.8-388.1)	**<0.001**
Tea drinking, N (%)				**0.011**
Non-drinking	2180 (68.0)	592 (62.3)	567 (62.7)	
Few times per month	91 (2.8)	31 (3.3)	28 (3.1)	
Few times per week	224 (7.0)	76 (8.0)	78 (8.6)	
Almost every day	710 (22.2)	251 (26.4)	232 (25.6)	
Alcohol drinking, N (%)				**<0.001**
Non-drinking	2274 (71.0)	622 (65.5)	579 (64.0)	
No more than once a month	119 (3.7)	59 (6.2)	56 (6.2)	
Once or twice a month	196 (6.1)	77 (8.1)	78 (8.6)	
Once or twice a week	209 (6.5)	68 (7.2)	70 (7.7)	
3-4 times a week	111 (3.5)	35 (3.7)	41 (4.5)	
Almost every day	296 (9.2)	89 (9.4)	81 (9.0)	
**BMI ≥ 24, N=3262**				
N	2004 (61.4)	672 (20.6)	586 (18.0)	
Energy intakes,kcal/d	2071 (1676-2496)	2124 (1772-2523)	2160 (1758-2660)	**<0.001**
Fat intakes, g/d	66.1 (47.2-90.1)	79.8 (56.9-101.6)	78.8 (56.8-108.2)	**<0.001**
Protein intakes, g/d	184.8 (147.2-230.8)	201.5 (163.6-254.4)	211.1 (167.2-261.4)	**<0.001**
Carbohydrate intakes, g/d	867.8 (690.7-1090.6)	813.2 (669.9-1016.0)	835.7 (652.5-1015.3)	**<0.001**
Vegetables intakes, g/d	321.1 (230.4-428.3)	287.4 (215.6-377.4)	291.4 (200.9-404.4)	**<0.001**
Tea drinking, N (%)				**<0.001**
Non-drinking	1338 (66.8)	376 (56.0)	346 (59.0)	
Few times per month	44 (2.2)	18 (2.7)	15 (2.6)	
Few times per week	130 (6.5)	67 (10.0)	43 (7.3)	
Almost every day	492 (24.6)	211 (31.4)	182 (31.1)	
Alcohol drinking, N (%)				**<0.001**
Non-drinking	1414 (70.6)	408 (60.7)	324 (55.3)	
No more than once a month	59 (2.9)	32 (4.8)	36 (6.1)	
Once or twice a month	138 (6.9)	53 (7.9)	54 (9.2)	
Once or twice a week	145 (7.2)	66 (9.8)	67 (11.4)	
3-4 times a week	79 (3.9)	34 (5.1)	37 (6.3)	
Almost every day	169 (8.4)	79 (11.8)	68 (11.6)	

IQR: interquartile range;
